# Consensus statement on the need for innovation, transition and implementation of developmental neurotoxicity (DNT) testing for regulatory purposes

**DOI:** 10.1016/j.taap.2018.02.004

**Published:** 2018-09-01

**Authors:** Ellen Fritsche, Philippe Grandjean, Kevin M. Crofton, Michael Aschner, Alan Goldberg, Tuula Heinonen, Ellen V.S. Hessel, Helena T. Hogberg, Susanne Hougaard Bennekou, Pamela J. Lein, Marcel Leist, William R. Mundy, Martin Paparella, Aldert H. Piersma, Magdalini Sachana, Gabriele Schmuck, Roland Solecki, Andrea Terron, Florianne Monnet-Tschudi, Martin F. Wilks, Hilda Witters, Marie-Gabrielle Zurich, Anna Bal-Price

**Affiliations:** a IUF – Leibniz Research Institute for Environmental Medicine, Duesseldorf, Germany; b University of Southern Denmark, Harvard T.H. Chan School of Public Health, USA; c Neurotoxicologist, Durham, NC, USA; d Albert Einstein College of Medicine Bronx, New York, USA; e Bloomberg School of Public Health, Founding Director (Emeritus) of Center for Alternatives to Animal Testing (CAAT), Johns Hopkins University, Baltimore, USA; f Finnish Centre for Alternative Methods (FICAM), University of Tampere, Tampere, Finland; g National Institute for Public Health and the Environment, RIVM Center for Health Protection, Bilthoven, Netherlands; h Centre for Alternatives to Animal Testing (CAAT), Johns Hopkins University, Baltimore, USA; i The Danish Environmental Protection Agency (EPA), Copenhagen, Denmark; j Department of Molecular Biosciences, School of Veterinary Medicine, University of California Davis, Davis, USA; k CAAT – Centre for Alternatives to Animal Testing, University of Konstanz, Konstanz, Germany; l Neurotoxicologist, Durham, USA; m Austrian National Coordinator of Testing Methods, Austria; n RIVM Center for Health Protection, Bilthoven and Institute for Risk Assessment Sciences, Utrecht University, Utrecht, Netherlands; o Organisation for Economic Co-operation and Development (OECD), Paris, France; p Bayer, Wuppertal, Germany; q Federal Institute for Risk Assessment (BfR), Berlin, Germany; r European Food Safety Authority (EFSA), Parma, Italy; s Department of Physiology, University of Lausanne and SCAHT, Switzerland; t SCAHT – Swiss Centre for Applied Human Toxicology, University of Basel, Basel, Switzerland; u VITO, Flemish Institute for Technological Research, Unit Environmental Risk and Health, Belgium; v European Commission –DG Joint Research Centre (JRC), Ispra, Italy; w Global Food Ethics, Johns Hopkins University, Baltimore, USA

**Keywords:** Developmental neurotoxicity, *In vitro* testing, Regulatory purposes

## Abstract

This consensus statement voices the agreement of scientific stakeholders from regulatory agencies, academia and industry that a new framework needs adopting for assessment of chemicals with the potential to disrupt brain development. An increased prevalence of neurodevelopmental disorders in children has been observed that cannot solely be explained by genetics and recently pre- and postnatal exposure to environmental chemicals has been suspected as a causal factor. There is only very limited information on neurodevelopmental toxicity, leaving thousands of chemicals, that are present in the environment, with high uncertainty concerning their developmental neurotoxicity (DNT) potential. Closing this data gap with the current test guideline approach is not feasible, because the *in vivo* bioassays are far too resource-intensive concerning time, money and number of animals. A variety of *in vitro* methods are now available, that have the potential to close this data gap by permitting mode-of-action-based DNT testing employing human stem cells-derived neuronal/glial models. *In vitro* DNT data together with *in silico* approaches will in the future allow development of predictive models for DNT effects. The ultimate application goals of these new approach methods for DNT testing are their usage for different regulatory purposes.

## Highlights

• An increased prevalence of neurodevelopmental disorders in children is observed.
• There is very limited information on neurodevelopmental toxicity (DNT) induced by environmental chemicals.
• A new framework is required for assessment of chemicals with the potential to disrupt brain development.
• *In vitro* DNT data together with *in silico* approaches should be used for regulatory purposes.

There is no doubt that an optimally developed brain is a profound asset to human functioning as well as for the sustainability and intellectual depth of any society. The human brain is by far the most advanced and complex brain in the animal world with regard to both its structure and functionality. Although brain development in all vertebrate species starts from a small tube of neuroectoderm, human embryonic brain development proceeds in a more complex manner than any other species. The human brain develops through a series of developmental stages that must occur in a particular sequence and at the right time. The final outcome on the human brain is critically dependent upon the physiology of these processes, each of which might be vulnerable to adverse effects from exposures to environmental chemicals or drugs.

A report by the US National Research Council (NRC) clearly documents the challenges in using rodent-based testing in general, to predict adverse outcomes in humans (NRC, 2007). Given extensive interspecies differences in brain morphology and function, based on specific cellular and molecular characteristics (Gassmann et al., 2010; Harrill et al., 2011; Baumann et al., 2016; Dach et al., 2017), and the paucity of chemicals tested for DNT (Makris et al., 2009; Tsuji and Crofton, 2012; Tohyama, 2016) the current reliance on DNT testing in rodents to predict effects in humans is called into question. While the US Environmental Protection Agency (OPPTS 870.630) and the Organization for Economic Co-operation and Development (OECD TG426) DNT study guidelines exist (US EPA, 1998; OECD, 2007), including guidance for interpreting DNT data in support of a pesticide registration developed by the US EPA and Health Canada (Moser et al., 2016), no routine testing for DNT is carried out in the U.S., in the EU, or elsewhere, as DNT testing is not required by law unless triggered by neurotoxic or endocrine effects in adult rodents. These triggers may not be sufficient (Bal-Price et al., 2018a) as some neurodevelopmental processes are not present in the adult brain (Fritsche et al., 2017). At the same time, performance of the DNT *in vivo* guideline studies involves the use of large numbers of animals and is therefore cost- and time-intensive (Crofton et al., 2012; Tohyama, 2016). These facts may contribute to the reason why only a few environmental substances (12 in total) are classified as human developmental neurotoxicants (Evans et al., 2016; Grandjean and Landrigan, 2006; Bjorling-Poulsen et al., 2008).

As a consequence of the large knowledge gaps on possible neurodevelopmental toxicity of chemicals, it is highly uncertain whether we are adequately protecting the health of our children's brains. This issue has been recently discussed among scientists and they came to the conclusion that a new framework must be adopted for assessing chemicals that have the potential to disrupt brain development and control the use of those that may pose a risk (Bennett et al., 2016; OECD, 2017; Fritsche et al., 2017).

The rise in neurodevelopmental impairments (*e.g.*, autism, cognitive functions, ADHD, dyslexia) among infants and children has been suspected to be caused by chemicals and/or other stressors including nutrition, stress, and gene-environment interactions (Julvez and Grandjean, 2013; Grandjean and Landrigan, 2014; McDonald and Paul, 2010; Grandjean et al., 2017; Washington State Departments of Ecology and Health., 2009).

It is now well accepted that humans have the potential to be exposed to thousands of man-made compounds (Egeghy et al., 2016; Isaacs et al., 2016) and the fact that only a small percentage of environmental contaminants have been tested for DNT, results in a critical and urgent need to develop a cost-efficient standardized alternative approach. Many methods are now available or under development both *in vitro* and *in silico*, that enable the study of human-specific molecular and cellular effects of chemicals, and their integration into *in silico* predictive models for developmental neurotoxicity (*e.g.*, *in vitro* neuronal/glial models derived from human stem cells, (Q)SARs, read-across and *in silico* models) (Bal-Price et al., 2015; van Thriel et al., 2012; Aschner et al., 2017;OECD, 2017).

Due to the existing deficiencies in knowledge on the DNT potential of many thousands of chemical compounds (NRC, 1984; Makris et al., 2009; Tohyama, 2016), which for practical reasons cannot be overcome by the current regulatory DNT guideline studies, initiatives are on-going that promote the development of time- and cost-effective integrative DNT strategies for regulatory purposes (Bal-Price et al., 2012, Bal-Price et al., 2015, Bal-Price et al., 2018a; Crofton et al., 2014; Smirnova et al., 2014; Schmidt et al., 2017). Though many reservations still exist precluding a straightforward replacement of animal testing, available data from cost- and time-efficient *in vitro* and lower organism models are already available, calling for consideration of their regulatory application and acceptance. With an emerging scientific consensus, regulatory initiatives are currently ongoing (OECD, 2017) under an OECD remit with the commitment of implementing concepts and tools needed to identify DNT hazardous substances. Identifying reliable and efficient *in vitro* and *in silico* methods in line with the 3R principles (Replacement, Reduction and Refinement) (Russell and Burch, 1959) to identify and characterize DNT hazards for regulatory purposes is of high priority to predict human health concerns, as the majority of chemicals have not yet been tested for their DNT potential (Makris et al., 2009; Tohyama, 2016).

One prominent example of successful use of an alternative model for human hazard evaluation is the epidemic of microcephaly that occurred in Brazil. Zika virus (ZIKV) infection was soon identified as a likely culprit, but it also appeared that ZIKV infection could not be detected in all microcephaly cases and that the infection did not uniformly result in adverse effects on brain development (Albuquerque et al., 2016). However, the molecular mechanisms leading to microcephaly induced by ZIKV infection, recently substantiated in a case control study (de Araújo et al., 2017), were clearly evidenced using human neurospheres derived from human induced pluripotent stem cells (hiPSCs) (Garcez et al., 2017; Dang et al., 2016). Application of such human cell-based *in vitro* approaches for assessing chemicals that could potentially affect the developing brain seems logical.

A recent approach illustrates how *in vitro* and *in silico* bioactivity data can be combined with kinetics modeling to derive ‘risk’ estimates' (Sipes et al., 2017). This approach, not yet applied to DNT, allows rapid estimations of possible interactions of chemical molecules with biological targets at environmentally relevant exposure levels. Such computational predictive approaches are highly attractive alternatives, as they can handle and integrate massive amounts and diverse types of information to facilitate toxicity predictions at the level of complexity of the intact human. In its report on Toxicity Testing in the 21st Century, the National Research Council (NRC, 2007, McCray et al., 2010), already 11 years ago called for the development of new approaches to human health risk assessment that would rely on mechanistic understanding of toxicity pathways using human *in vitro* models and computer-based modeling, rather than apical animal testing (Krewski et al., 2010). Knudsen et al. (2013) reiterated the recommendation that *in silico* approaches should be included in future toxicity assessments.

Progress for alternative DNT testing should be accelerated, in a manner fit for scientific and regulatory acceptance. *In vitro* screening programs have already been initiated. For example, the U.S. EPA ToxCast high-throughput screening assays program, where so far approximately 2000 chemicals have been examined for ~700 assay endpoints (Richard et al., 2016), and the US Federal Tox21 Program, which has screened nearly 8500 chemicals in about 50 selected assays (Tice et al., 2013; Attene-Ramos et al., 2013). Unfortunately, few assays in these programs are directly relevant to brain development. These issues highlight the critical need for a global strategy that stimulates the development and application of alternative models for DNT testing (Leist et al., 2014; Bal-Price et al., 2018b).

The present special issue contributes to defining the state-of-the-art of currently available alternative approaches for DNT evaluation (Bal-Price et al., 2018a), and it also outlines how traditional animal testing could be improved by alignment of rodent testing with effects seen in children after neurotoxic exposures (Vorhees, 2018). The potential alternative approaches relevant to DNT testing are discussed including key neurodevelopmental processes and AOP-driven integrated approaches to testing and assessment (IATA) for DNT screening and prioritization (Bal-Price et al., 2018a, Sachana et al., 2018), application of non-mammalian models such as zebrafish (Geier et al., 2018; Padilla et al., 2018), developing a systems biology approach and an ontology-driven animal-free test battery for DNT testing (Hessel et al., 2018), the role in toxicant sensitivity of alterations in cellular metabolism during human neurogenesis (Delp et al., 2018) and a molecular species comparison using methods such as the ‘Neurosphere Assay’ (Masjosthusmann et al., 2018).

Accordingly, the authors of this statement, which include stakeholders from regulatory agencies, academia and industry, and some of whom have contributed a manuscript to this special issue, recommend to advance rigorously the science and regulation related to DNT testing and the implementation of reliable animal-free human-focused mechanistic DNT hazard assessment by:

1. Expanding the data requirements and implementation of an alternative testing strategy for DNT testing in current regulatory frameworks for industrial chemicals including pesticides, biocides and other chemical substances occurring in our food and environment in order to adequately protect the healthy development of our children.
2. Developing and adopting a new OECD guidance for assessing chemicals that have the potential to disrupt human brain development. Here, DNT hazard characterization and prioritization for regulatory purposes through reliable and efficient *in vitro* and *in silico* methods in line with the 3R principles in an IATA framework is of high priority. Such alternative DNT testing should be based on test methods assessing compounds' effects on key neurodevelopmental events relevant to human biology.
3. Populating this framework with data, through the development of a cost-efficient, standardized and holistic alternative approach for screening large numbers of environmental contaminants to identify those that may pose a DNT risk. Data generated by this framework should also be leveraged to refine *in silico* approaches with the goal of developing reliable models predictive for human DNT effects.
4. Considering implementation of alternative DNT testing models that are already available for regulatory applications. Efforts should be put towards developing criteria to determine readiness and the acceptability of such methods and additional guidance for how the outcome of those tests should be evaluated in the regulatory context.
5. Using *in vitro* mechanistic DNT data to facilitate development of computational predictive approaches that can handle and integrate massive amounts and diverse types of information. Including kinetic quantitative *in vitro* to *in vivo* extrapolation models (QIVIVE) would facilitate toxicity predictions of likely safe human exposure levels. Yet, experimental data is needed to inform and validate such mathematical models.
6. Closing remaining knowledge gaps through further research with the goal of supporting more effective regulation. There is agreement in the *DNT Community* (Bal-Price et al., 2015, Fritsche et al., 2017) on the necessity for further international research on testing strategies, which will require funding by all stakeholders, including government, non-government organizations and industry.

We envisage that the present DNT special issue of TAAP on Alternative Approaches to Developmental Neurotoxicity (DNT) Evaluation will inspire both scientists and regulators to follow these recommendations and to develop rational and more efficient approaches to identify chemical exposures that may pose a risk to the developing brains of future generations.

## Transparency document

Transparency document. Image 1
